# The Effect of Dietary Inclusion of Microalgae *Schizochytrium* spp. on Ewes’ Milk Quality and Oxidative Status

**DOI:** 10.3390/foods11192950

**Published:** 2022-09-21

**Authors:** Foivos Zisis, Panagiota Kyriakaki, Fotis F. Satolias, Alexandros Mavrommatis, Panagiotis E. Simitzis, Athanasios C. Pappas, Peter F. Surai, Eleni Tsiplakou

**Affiliations:** 1 Laboratory of Nutritional Physiology and Feeding, Department of Animal Science, School of Animal Biosciences, Agricultural University of Athens, Iera Odos 75, 11855 Athens, Greece; 2 Laboratory of Animal Breeding & Husbandry, Department of Animal Science, Agricultural University of Athens, Iera Odos 75, 11855 Athens, Greece; 3 Vitagene and Health Research Centre, Bristol BS4 2RS, UK

**Keywords:** DHA fatty acid, microalgae, sheep, functional food, rumen, antioxidants

## Abstract

An unprecedented challenge for nutritionists arises during the 21st century in order to produce highly nutritious and functional food which promotes human health. Polyunsaturated fatty acids (PUFA) that are highly contained in microalgae have broadly been confirmed for preventing cardiovascular diseases and regulating immune-oxidative status. However, their optimum dietary inclusion level needs to be defined since PUFA are prone to oxidation. For this purpose, 24 cross-bred dairy ewes, were separated into four groups (n = 6) and were fed with different levels of microalgae *Schizochytrium* spp. [0 (CON, no microalgae), 20 (SC20), 30 (SC30) and 40 (SC40) g/ewe/day] for 60 days. The results showed that although the production parameters were not impaired, milk fat content was decreased in medium and high-level supplemented groups while protein content was suppressed only for the medium one. Concerning the fatty acids (FA) profile, the proportions of C_14:0_, trans C_18:1_, trans-11 C_18:1_, cis-9, trans-11 C_18:2_, trans-10, cis-12 C_18:2_, C_20:5_ (EPA), C_22:5n-6_ (DPA), C_22:6n-3_ (DHA), the total ω3 FA and PUFA were significantly increased, while those of C_18:0_, cis-9 C_18:1_ and C_18:2n-6c_ were decreased in the milk of treated ewes. Additionally, in the S40 group an oxidative response was induced, observed by the increased malondialdehyde (MDA) levels in milk and blood plasma. In conclusion, the dietary inclusion of 20 g *Schizochytrium* spp./ewe/day, improves milks’ fatty acid profile and seems to be a promising way for producing ω3 fatty acid-enriched dairy products.

## 1. Introduction

Sheep milk as well as dairy products contain prominent levels of saturated fatty acids (SFA), which increase the risk of incidence of coronary heart disease (CHD) [1]. In contrast, polyunsaturated fatty acids (PUFA), also contained in sheep milk, have extensively been researched for preventing inflammation, atherosclerosis, thrombosis, and other cardiovascular diseases [2,3] and for brain development [4]. Subsequently, it is of utmost importance for nutritionists to find innovative ways to produce food and products with high PUFA content [5], including omega-3 fatty acids, in environmentally sustainable ways.

Likewise, microalgae containing a substantial amount of fatty acids particularly valuable to human nutrition, such as docosahexaenoic (DHA) and docosapentaenoic (DPA) could be used for the enrichment of milk and dairy products with PUFA, including omega-3 fatty acids. More specifically, the supplementation with *Schizochytrium* spp. in ruminants’ diet, a microalga highly contained in ω-3 PUFA, such as DHA [6], has already been confirmed as an efficient nutritional strategy for milk enrichment with PUFA in cows [7], goats [8,9] and ewes [10,11].

Notwithstanding, the enclosure of PUFA in ruminants’ diets may result in suppression of the rumen biohydrogenation procedure promoting the formation of intermediates isomers of C_18:2_, for instance, trans-10, cis-12 C_18:2_ and trans-10 C_18:1_ [12]. This is an indication of potential inhibition of milk fat synthesis [13,14], a phenomenon described as milk fat depression (MFD) which severely impairs the dairy industry. The size of the phenomenon depends on the species [15], the ratio of fiber/starch diets [14,16] and the level and profile of PUFA inclusion [8].

Nevertheless, PUFA and notably n-3 LC-PUFAs are particularly susceptible to oxidation, enzymatic and non-enzymatic [17], due to the number of double bonds and their spot in the fatty acid chain [18]. The result is the production of metabolites and Reactive Oxygen Species (ROS), the concentration of which indicate whether oxidative stress takes place [17]. Oxidative stress (OS) is the result of the imbalance between oxidant and antioxidant compounds [19] which induces damage to cells and tissues [20] and might also cause some detrimental changes in the nutritional value and quality of food products [17]. The source of PUFA along with the level of inclusion can both affect the oxidative status of several tissues, as has already been evinced in rats [21,22] and goats [23]. However, scarce evidence exists about the optimum inclusion level of PUFA from microalgae in ewes’ diets.

Besides, *Schizochytrium* spp. is rich in antioxidant compounds [24], such as phenols, carotenoids, etc. [25], a feature with pivotal importance in balancing the oxidative status in PUFA supplemented goats [23]. However, it has been suggested that goats and sheep respond diversely to oxidative stimulus [26]. Although the impact of *Schizochytrium* spp. on oxidative status of both organism and milk has adequately been documented in goats [23,27], there is only limited information on dietary manipulation of ewe milk composition which is of enormous socio-economic importance within the Mediterranean basin.

Lastly, even though there is research evidence regarding the effect of *Schizochytrium* spp. in ruminant nutrition, the transfer efficiency of specific, beneficial fatty acids in milk is orchestrated by several factors, such as lactation stage, the whole diet composition, breed, host-microbiome interactions, etc. Taking into consideration the aforementioned issues, the enrichment of milk and dairy products with PUFA, including omega-3 fatty acids, should be simultaneously investigated by the preservation of their oxidative status under a holistic perspective since the production of functional foods requires the implementation of precision feeding strategies. The objective of our study was to investigate the effects of dietary inclusion of *Schizochytrium* spp., at three different levels (20, 30 and 40 g/ewe/day), first and foremost on ewes’ milk quality and oxidative stability.

## 2. Materials and Methods

### 2.1. Animals and Diets

Twenty-four crossbred dairy ewes [Lacaune x Local (Greek) breed] of comparable age (3–4 years old), at middle-late lactation (150 ± 10 days in milk) were used. The experiment was carried out at an affiliate experimental-constructed farm part of the “Cooperative of Kalavryta” under traditional conditions and local ways of production. The ewes were separated into four homogenous groups (n = 6 per group) according to their milk yield (Fat Corrected Milk, FCM 6%), body weight and age. The animals were grouped fed into six place feeders. The available feeding space was higher than the one recommended for adult housed ewes (0.33 m per animal) in order to favor simultaneous access and lower competitive interactions at the feeder among animals. Forage was provided with the concentrate in two equal portions after milking while no concentrate was offered in the milking parlor. Diet consumption was recorded on a daily basis ensuring no refusals occurred. The daily intake for each ewe was 1.83 kg alfalfa hay and 1.83 kg concentrates, throughout the experimental period (F:C = 50:50). The concentrates supplied to the control (CON) group had no microalgae while those of the treated groups were supplemented daily with 20 (SC20), 30 (SC30) and 40 (SC40) g *Schizochytrium* spp. Thus, the microalgae daily intake was 20 g, 30 g and 40 g/ewe in SC20, SC30 and SC40 groups, respectively. The three different doses of *Schizochytrium* spp. were incorporated into the same concentrates as that of the control group, substituting soybean oil and plant fat in order to achieve comparable diets (Table 1). The supplemented *Schizochytrium* spp. used was a commercial product (DHA GOLD S17-B, DSM Nutritional Products, Marousi, Greece). The concentrate diets were prepared once a month and kept under the appropriate conditions in order to prevent any oxidation of the microalgae. The experimental period lasted 60 days while the first 10 days were considered an adaptational period.

### 2.2. Samples Collection

#### 2.2.1. Feed Analysis

Alfalfa hay and concentrates dry matter (Official Method 7.007) and crude protein (Official Method 7.016) were analyzed according to AOAC (1984). In addition, ether extract was evaluated according to Soxhlet [28], and neutral detergent fiber (NDF) in addition to acid detergent fiber (ADF) was determined according to Van Soest et al. [29]. FA profile of feed samples was also analyzed according to the method of O’Fallon et al. [30].

#### 2.2.2. Milk Collection

The ewes were milked twice a day at 07:30 and 19:30 by a portable milking machine. Individual milk samples were collected into plastic tubes at 0, 10, 20, 30, 40 and 50 days of the main experimental period for the determination of chemical composition, fatty acid profile, total antioxidant content and antioxidant enzymes activities after pooling the yield from the evening and the morning milking. The milk samples were stored at −80 °C, before the analysis of the fatty acid profile and enzyme activity.

##### Milk Chemical Composition

Milk samples were analyzed for fat, protein, lactose, total solids and solid not fat by IR spectrometry (MilkoScan 120; Foss Electric, Hillerød, Denmark) after proper calibration according to Gerber (British Standards Institution, 1955) and Kjeldahl (International Dairy Federation, 1993).

#### 2.2.3. Blood Plasma Preparation

The blood samples were collected from the jugular vein and put into 17 Units/mL heparin-containing tubes (BD Vacutainer, Plymouth, UK) at 0, 10, 20, 30, 40 and 50 days of the main experimental period for the determination of the total antioxidant content and antioxidant enzymes activities. After the collection, the blood samples were subsequently centrifuged at 2700× *g* for 10 min to separate plasma from the cells. After, the blood samples were stored at −80 °C, prior to enzyme activities analysis.

### 2.3. Enzyme Assays, Oxidative Stress Biomarkers and Total Antioxidant Capacity

The measurement of the enzyme activity (SOD, CAT, GR, GSH-Px), the oxidative stress biomarkers (MDA, PC) and the total antioxidant capacity (FRAP, ABTS) were based on the principles of spectrophotometry and conducted as described below.

#### 2.3.1. Oxidative Stress Biomarkers

The measurement of the malondialdehyde (MDA), a final product of lipid peroxidation, was performed as described by Nielsen et al. [31]. For the assay conducted, 1 mL of non-skimmed milk was included in a solution consisting of 7 mL of 1% orthophosphoric acid and 2 mL of 0.6% thiobarbituric acid (TBA). After, the mixture heating for 60 min at a temperature of 100 °C, the samples were filtrated through a cellulose acetate microfilter (0.45 μm) and after that, each sample’s absorbance was recorded at 532 nm. On the other hand, for the blood samples, 100 μL of the sample was used for inclusion in a solution of 700 μL of 1% ortho-phosphoric acid and 200 μL of 0.6% thiobarbituric acid (TBA, Sigma-Aldrich). After, sample incubation at 100 °C for 60 min and cooling, their absorbance was also recorded at 532 nm.

The protein carbonyls (PC) assay was performed by the method established by Patsoukis et al. [32] for blood plasma with some adjustments on the amount of the milk samples. Firstly, a solution of 50 μL of TCA (trichloroacetic acid, Sigma-Aldrich, CO, USA) was mixed with 50 μL of plasma and/or milk. After that, there was an incubation in ice for 15 min with following centrifugation at 15,000× *g* for 5 min. Then, the supernatant was excluded and 500 μL of DNPH (2,4-dinitrophenylhydrazine, Sigma-Aldrich, CO, USA) was added to the formed pellet. Then the samples were left for incubation for 1h (in dark) with vortexing every 15 min. After 1h incubation samples were centrifugated at 15,000× *g* for 5 min at 4 °C. Then again, the supernatant was removed, and 1 mL of TCA was added, vortexed and centrifuged (15,000× *g*/5 min/4 °C). Once more, the supernatant was removed and 1 mL of ethanol–ethyl acetate (Merck KGaA) was added, vortexed and centrifuged (15,000× *g*/5 min/4 °C). At the last phase of the assay, the supernatant was removed and 1 mL of 5 M urea (pH = 2.3) was added, before the samples vortexed and incubated at 37 °C for 15 min. After that, the samples were centrifugated (15,000× *g*/3 min/4 °C) and absorbance was measured at 375 nm.

#### 2.3.2. Antioxidant and Free Radical Scavenging Activity

The antioxidant capacity and free radical scavenging activity were performed by the Ferric Reducing Antioxidant Power (FRAP) assay and the results were expressed as μΜ ascorbic acid. Besides, the total antioxidant capacity was monitored with ABTS (2,2’-azino-bis (3 ethylbenzthiazoline-6-sulfonic acid) radical scavenging capacity. Both assays were performed as previously described by Tsiplakou et al. [33].

#### 2.3.3. Assays for Antioxidant Enzymes

The evaluation of the activity of catalase (CAT), glutathione peroxidase (GSH-Px) and superoxide dismutase (SOD) in both milk and blood plasma as well as for glutathione reductase (GR) and glutathione transferase (GST) in blood plasma were performed according to the methods described by Tsiplakou et al. [33].

### 2.4. Milk FA Determination

The fatty acid profile of milk samples was also examined according to the method of Nourooz-Zadeh and Appelqvist [34] as referred to by Tsiplakou et al. [35]. Briefly, an 8 mL milk sample was transferred into a 50 mL falcon tube, where 15 mL of isopropanol was added. After vigorous shaking, 12.5 mL hexane was added, and the mixture was shaken. After centrifugation, the upper layer was transferred to a new falcon. The lower layer was extracted twice with 12.5 mL hexane, and the supernatants were pooled with the previous hexane layer. After the addition of 7.5 mL of 0.47 M aqueous Na_2_SO_4,_ the hexane layer was collected into a pyrex test tube and evaporated. Then, in 40 mg of lipid, 2 mL hexane was added followed by 40 μL of methyl-acetate. After vortexing, a 40 μL methylation reagent (1.75 mL methanol/0.4 mL of 5.4 mol/L sodium methylate) was also added. The mixture was vortexed and allowed to react for 10 min, then 60 μL of termination reagent (1 g oxalic acid/30 mL diethylether) was added. The sample was then centrifuged for 5 min at 2400× *g* and the liquid layer was removed and used directly for chromatographic determination. For the determination of FAs, an Agilent 6890 N supplied with an HP-88 capillary column (60 m × 0.25 mm i.d. with 0.20 μm film thickness, Agilent) and a flame ionization detector (FID) was used. The temperature settings of the FID were established at 260 °C by placing a set of instructions at 120 °C held for 1 min. After this, two stages of 3 min each ensued, one of 1.25 °C/min to 230 °C, and the other of 10 °C/min to 240 °C. The carrier gas used was Helium with the linear velocity set at 30 cm/s. For peaks to be detected and quantified a 37-component FAME mix standard (Supelco, Sigma-Aldrich, St. Louis, MO, USA) was used. Moreover, extra standards were used for the C_18:2 cis-9_, _trans-11_, C_18:2 trans-10_, _cis-12_ and C_18:1 trans-11_ FA (Sigma Adrich Co., St. Louis, MO, USA). A heptadecanoic methyl ester (C17:0) was used as an internal standard for the chromatographic analysis (Fluka, Sigma, Aldrich) for the milk samples. The FA were grouped according to Mavrommatis et al. [27]:

Short-Chain Saturated Fatty Acids (SCFA) = C_6:0_ + C_8:0_ + C_10:0_ + C_11:0_, (1)

Medium-Chain Saturated Fatty Acids (MCFA) = C_12:0_ + C_13:0_ + C_14:0_ + C_15:0_ + C_16:0_, (2)

Long-Chain Saturated Fatty Acids (LCFA) = C_17:0_ + C_18:0_ + C_20:0_, (3)

Mono-Unsaturated Fatty Acids (MUFA) = C_14:1_ + C_15:1_ + C_16:1_ + C_17:1_ + _cis-9_ C_18:1_ + _trans-11_ C_18:1_ + _trans_ C_18:1_, (4)

Poly-Unsaturated Fatty Acids (PUFA) = _cis-9, trans-11_ C_18:2_ + C_18:2n-6c_ + C_18:2n-6t_ + C_18:3n-3_ + C_18:3n-6_ + C_20:3n-3_, (5)

Saturated Fatty Acids (SFA) = SCFA + MCFA + LCFA,(6)

Unsaturated Fatty Acids (UFA) = PUFA + MUFA,(7)

Saturated/Unsaturated (S/U) = (SCFA + MCFA + LCFA)/(PUFA + MUFA),(8)

Atherogenic index (AI) = (C_12:0_ + 4 × C_14:0_ + C_16:0_)/(PUFA + MUFA), (9)

Thrombogenic index (TI) = (C_14:0_ + C_16:0_ + C_18:0_)/(0.5 × MUFA) + (0.5 × n-6 PUFA) + (3 × ω3 PUFA) + (ω3 PUFA/ω6 PUFA), (10)

Health promoting index (HPI) = (ω6 PUFA + ω3 PUFA + MUFA)/(C_12:0_ + 4 × C_14:0_ + C_16:0_). (11)

### 2.5. Statistical Analysis

The statistical experimental analysis was performed using the SPSS statistical software (v 26.0 IBM Corp., Armonk, NY, USA) and the experimental data are demonstrated as mean ± standard error of means (SEM). The effect of the dietary treatment among the four groups was evaluated using a general linear model (GLM) for repeated measures analysis of variance (ANOVA). For this analysis, the dietary treatments (CON, SC20, SC30 and SC40) were defined as the fixed factor while the sampling time (*T*) was the repeated measure. The interaction between dietary treatment and sampling time (*D × T*) was also evaluated in accordance with the following model:

(12) $$Y_{ijkl}=\mu +D_{i}+T_{j}+A_{k}+{\left(D\times T\right)}_{ij}+e_{ijkl}$$

where *Y_ijkl_* is the dependent variable, *μ* the overall mean, *D_i_* the effect of dietary treatment (*i* = 4; CON, SC20, SC30, SC40), *T_j_* the effect of sampling time (*j* = 6; 10, 20, 30, 40, 50), *A_k_* the ewes’ random effect, (*D* × *T*)*_ij_* the interaction between the dietary treatment and the sampling time and *e_ijkl_* the residual errors. Measurement at day zero (0) was considered as covariance aiming to balance any fluctuation amongst the dietary groups.

For all the statistical tests, significance was set at 0.05.

## 3. Results

### 3.1. Ewe Performance and Milk Composition

The dietary incorporation of *Schizochytrium* spp., independent of its levels, did not significantly affect the milk performance (Table 2). Milk yield also remained identical between the start and the end of the experiment; thus, the dry matter intake and body weight did not change (data not shown). Concerning milk chemical composition, milk fat (Fat%) was substantially decreased in the treated groups (*p* < 0.01). In detail, milk fat from SC30 was substantially decreased compared to the CON group; milk fat from SC40 was substantially decreased compared to the CON and SC20 group. Moreover, total solids (TS%) were significantly decreased among the dietary treatment groups (*p* < 0.01). More specifically, TS% in the SC30 group was shown to decline in comparison to CON and SC40. However, there was no significant interaction between the two factors, dietary treatment, and sampling time during the trial, as depicted in Table 2. Milk yield, fat yield, FCM, ECM, protein, protein yield, lactose and SNF were not significantly modified (Table 2).

### 3.2. Milk Fatty Acids Profile

The incorporation of *Schizochytrium* spp. in the ewes’ diets did not modify the proportions of SFA, UFA and MUFA among the four different levels, as presented in Table 3. Regarding the time effect, the proportion of SFA decreased, while that of UFA and MUFA increased throughout the experimental period (*p* < 0.001). Additionally, significant interactions (D × T) for UFA, SFA, and MUFA were also observed (*p* < 0.01). In contrast, the proportion of PUFA substantially increased in SC20 and SC30 groups compared to the CON while the uppermost was for SC40 compared to the other groups. Moreover, the proportion of LCFA showed a sharp decline as a result of increasing inclusion dietary level of *Schizochytrium* spp. On the other hand, the proportion of SCFA was not affected by the dietary inclusion level of *Schizochytrium* spp. but showed a linear decline throughout the experimental period (*p* < 0.001). Furthermore, the proportion of MCFA was significantly higher in the SC30 and SC40 groups compared to the CON (*p* < 0.05), while the lowest value was observed in the 50th compared to the 10th and 20th experimental day (*p* < 0.001).

The proportion of stearic acid (C18:0) was significantly increased in the treated groups, with the lowest values to be observed in the SC30 and SC40 groups compared to the SC20 and CON fed ewes, whilst its content in the SC20 group compared to the CON one was also reduced. On the contrary, significant enhancement was reported from the 30th day and onwards. Moreover, the proportion of trans C18:1 and the vaccenic acid were increased in the milk of the treated groups (*p* < 0.001). On the other hand, a sharp decrease (*p* < 0.001) was observed in the proportion of oleic acid (_cis-9_ C18:1) which was more intense in the higher inclusion levels of *Schizochytrium* spp. (SC30 and SC40). A notable increase in the proportion of _cis-9_ C_18:1_ occurred from the 20th experimental day onwards (*p* < 0.001), which resulted in a modest interaction (*p* < 0.05). The proportion of C_18:1 cis-11_ showed an increasing trend in the SC20 and SC40 groups compared to the CON fed ewes (*p* < 0.1). Furthermore, an escalated decline in the proportion of linoleic acid (C_18:2 n-6 cis_) in the milk of treated groups was found (*p* < 0.001). However, only a minor increase occurred from the 30th day which resulted in no further interaction. Linolelaidic acid (C_18:2n6t_) and CLA (_cis-9, trans-11_ C_18:2_) on the other hand, showed a significant increase in the milk of *Schizochytrium* spp. fed ewes (*p* < 0.001). In addition, _trans-10, cis-12_ C_18:2_ (CLA isomer) portrayed a linear increase for the groups fed with the microalgae. The proportion of nervonic acid (C_24:1_) highly increased in the milk of SC40 and SC30 ewes (*p* < 0.001). Regarding the proportions of DPA (C_22:5n6_) and DHA (C_22:6n3_), a substantial increase was found in the milk of SC30 and SC40 ewes (*p* < 0.001). Additionally, the proportion of the DHA was different in the SC20 milk compared to the other dietary groups.

The transfer efficiency of DPA, from feed to milk, was 9.5, 7.4, and 7.8% for SC20, SC30, and SC40 groups, respectively (data not shown). In addition, DHA resulted in a much higher transfer efficiency for the SC30 group (30.3%), while the lowest was for the SC40 group (22%) (data not shown).

### 3.3. Blood Plasma and Milk Oxidative Status

The total antioxidant capacity, antioxidant enzyme activities, oxidative status of both blood plasma and milk, and oxidative stress indicators/indexes are presented in Table 4. The MDA and PC contents were found to be increased significantly (*p* < 0.05) in the blood plasma of treated ewes. The total antioxidant capacity measured by the ABTS assay increased significantly (*p* < 0.01) in the blood plasma of SC40 ewes, while it did not differ among the dietary treatments when determined by the FRAP assay. From the 20th experimental day onwards the ABTS values were significantly increased (*p* < 0.001). In blood plasma a trend for increase in CAT activity (*p* < 0.1) in the SC30 compared to the CON and SC20 and in GR activity in the SC20 compared to the CON ewes were found. Additionally, a significant enhancement in the GST activity (*p* < 0.05) of the SC30 and SC40 compared to the CON ewes was observed. Both GST and CAT activities were significantly enhanced from the 30th experimental day onwards (*p* < 0.01).

Moreover, the milk analysis depicted noteworthy adjustments for the MDA and the PCs concentration level, as for the microalgae incorporation level concerns. In its entirety, MDA concentration was enhanced for the SC20 group compared to the CON, whereas it was also amplified for SC40 compared to the CON and SC30 (*p* < 0.01). Although there were significant lower MDA values for the 50th day compared to the 20th and the 40th (*p* < 0.05), there was no significant interaction of D × T. Besides, the lower concentration of PCs observed in the milk of the SC30 compared to the CON ewes (*p* < 0.05), their highest values were found in the 20th and lowest in the 50th day (*p* < 0.01), no further relation was established. Moreover, the total antioxidant capacity measured by the ABTS and FRAP assays, as well as the antioxidant enzyme activities (SOD, CAT, GSH-Px) were not affected by the dietary treatment. FRAP and ABTS values declined throughout the experimental period. Both ABTS and FRAP showed no interaction of D × T. Additionally, SOD presented an enhancement for the 40th and 50th days and marginal interaction for D × T (*p* < 0.05). GSH-Px portrayed also a continuous upsurge from the 20th to the 40th day but a major decline was also exhibited for the 50th day (*p* < 0.001).

## 4. Discussion

In a never-ending changing world, from the prospect of nutrition knowledge, the inclusion of microalgae *Schizochytrium* spp. in ewes’ diets might be a proper strategy for the production of eco-friendly and sustainable milk and dairy products. The purpose for the inclusion is not simply limited to the beneficial fatty acids’ enhancement but also to comply with the oxidative status of ewes and the oxidative stability of the derived dairy products. Due to the high amount of PUFA included in the microalgae diet, the outcome of oxidation could be severe, as stated before. The favorable effect, therefore, would be for animals to avoid oxidative stress and produce dairy products rich in ω-3 fatty acids of enlarged oxidative stability. Nonetheless, this research study could be a pivotal part of a collective effort for better quality dairy products with functional properties to be produced.

### 4.1. The Inclusion Level Affect Milk Composition

Although microalgae had a strong fish-like odor and the fat level of the concentrates was high, the dry matter intake was not affected throughout the experiment. In our previous study on goats [8] and in the study by Papadopoulos et al. [36] the daily supplementation with over 40 g/animal with *Schizochytrium* spp. declined the DMI majorly through the hypophagic effect on brain satiety centers [37]. Previous studies conducted on the dietary incorporation of *Schizochytrium* spp. in ruminants, resulted in various alterations in the milk characteristics. In-depth, the milk yield and the fat corrected milk (FCM6%) were not affected by the diets, which is in accordance with previous studies in ewes [10,11,36] and goats [8] fed the same microalgae containing diets. However, it has been commonly reported, as indicated beyond, that the dietary incorporation of feed constituents rich in PUFA may lead to alteration in the rumen biohydrogenation process and subsequently to a decrease in milk fat eliciting milk fat syndrome (MFD) [13]. In addition to that, our results indicate the milk fat decrease as a result of the incorporation level of microalgae at 30 g/kg in line with previous studies on ewes fed microalgae at 20 g/kg [11] and goats obtaining microalgae at 40 g/kg [8]. This outcome, however, could be attributable to the differences between the breeds (Lacaune x local), used in this experiment in contrast to the Assaf ewes used by Bichi et al. [11], the whole diet composition, and the stage of lactation [27]. Protein content was also suppressed in SC30 ewes exhibiting further differences of *Schizochytrium* spp. amongst sheep and goats where milk protein remained unaffected. However, it seems that ewe responses to milk protein suppression under PUFA rich algae are consistent with other comparable outcomes unveiled earlier [10,15]. Additionally, as far as the lactose content is concerned, there were no significant changes among the treatment groups.

### 4.2. Supplementation of Schizochytrium spp. Improved the Milk Fatty Acids Profile

The obtained results of this experiment support the primary hypothesis that dietary *Schizochytrium* spp. can enhance milk PUFA with ω-3 fatty acids. Furthermore, the inclusion of *Schizochytrium* spp. containing a high amount of PUFA is considered to be a good source of DHA as has previously been shown, making it an effective and safe alternative dietary additive for the improvement of milk and dairy products quality. The proportion of the short chain fatty acids was also not modified in ewes [11], goats [8] or cows [38] fed with *Schizochytrium* spp. On the other hand, the results for the medium chain fatty acids were in contrast with previous studies in cows [7]. More specifically, the proportions of both C_14:0_ and C_16:0_ increased [8,36] while only that of C_16:0_ decreased [10] in the milk of *Schizochytrium* spp.-fed animals.

The FA profile results, unambiguously imply that some conversions occurred in the last part of the rumen biohydrogenation process. Stearic acid (C_18:0_) is the final product of rumen biohydrogenation deriving from the transfiguration of vaccenic acid (C_18:1_ trans-11) [39]. Important to note is that the amplest intermediates in milk, formed in the rumen, are those of C_18:1_ trans and C_18:1 trans-11_, particularly, which constitute transitional fatty acids produced during the biohydrogenation process of linoleic and linolenic acid. The outcome for the C_18:0_ is in agreement with those data from previous studies on ewes [10,36] and goats [40] fed with microalgae. These findings are further supported by the increasing tendency for the total C_18:1_ trans FAs in milk. Moreover, the sharpest decrease in stearic acid which was accompanied by an increase in C_18:1_ trans in the milk of ewes fed with the highest level shows a dose-dependence inhibition in the rumen biohydrogenation process which has been also confirmed in a previous study by Mavrommatis et al. [27] in goats. In agreement with the findings over stearic acid is the decrease for the oleic acid (C_18:1 cis-9_), the most plentiful cis MUFA in milk fat [5]. The decreased abundance of stearic acid in the mammary gland might inhibit the utilization and de novo synthesis of oleic acid by the Δ-9 desaturase [41], with C_18:0_ operating as a substrate [42].

Likewise, vaccenic acid (C_18:1 trans-11_) was significantly increased among the treatment groups. In the usual course of events, in diets containing marine algae or fish oil, the increase in milk C_18:1_ trans is associated with the elevated concentration of C_18:1 trans-6_ to C_18:1 trans-15_ [5]. Additionally, in highly fermentable diets [43] or under low pH conditions in the rumen [13], changes in the normal biohydrogenation pathways might occur, inhibiting milk fat synthesis, such as the substitution of C_18:1 trans-11_ with C_18:1 trans-10_, resulting in the decline of biohydrogenation transition from 18C PUFA to the stearic acid [44].

Moreover, in this study, most of the increase in CLA cis-9, trans-11 (Table 3) is ascribed to the increase in the C_18:1_ trans-11. The CLA _cis-9, trans-11_ concentration was increased in the milk of the microalgae-fed ewes, since C_18:1_ trans-11 functions as a substrate for the formation of CLA _cis-9, trans-11_ in the mammary gland via the desaturation by Δ-9 desaturase on C_18:1 trans-11_ [45]. It is of the essence to mention that in order to increase milk CLA, rumen vaccenic acid concentration should be increased, making it available for endogenous synthesis in the mammary gland. Therefore, by the enhancement of the Δ-9 desaturase’s activity, more increase arises for CLA _cis-9, trans-11_ [46]. In general, marine algae is known to have an eminent impact on milk fat CLA _cis-9, trans-11_ concentrations on a g oil/kg diet DM basis compared to plant oil sources [47]. These observations already have been acknowledged to be of great importance for promoting viable and eco-friendly solutions to produce dairy products with valuable fatty acids for human health and consumer choice.

However, in our study a significant decrease in milk fat was depicted, implying the occurrence of fatty acids with an anti-lipogenic effect, which caused Milk Fat Syndrome (MFD), starting at the level of 30 g/kg *Schizochytrium* spp. inclusion. The MFD in a general term, caused by dietary supplementation, tends to decrease milk fat without compromising the milk yield or the other milk features [5]. Apart from C_18:2 cis-9_, _trans-11_, C_18:2 trans-10, cis-12_, the second plentiful intermediate isomer of rumen biohydrogenation, was also significantly amplified. Profoundly, this CLA isomer (C_18:2_, _trans-10, cis-12_) was substantially enhanced, with every group having a substantial difference from one another. As has been described by other studies, in diets fed with marine lipids, the increased presence of the C_18:2 trans-10, cis-12_ was linked to a plausible indication of MFD as an anti-lipogenic factor. However, the former cannot be completely confirmed since other anti-lipogenic factors and fatty acids intermediates may also trigger off MFD [48].

It is most certainly apprehensible by the FA profile results that the concentration of both DPA and DHA fatty acids in milk was elevated in a corresponding manner to the dietary inclusion level of *Schizochytrium* spp. The abundance of high-quality food rich in n-3 fatty acids is an important aspect of the extensive application, for this reason, the consumption of n-3 PUFA is acknowledged to affect various human health conditions, such as the regulation of vascular and cardiac hemodynamics, triglycerides, inflammation, thrombosis, and arrhythmia [2].

As far as the health point of view is concerned, the linear decrease in the atherogenicity index (AI) in the milk of treated ewes during the experimental sampling period, shows significant importance for human health and fulfills the aim described by the Dietary Guidelines for the Americans to reduce the intake of SFAs and replace them with MUFA and PUFA instead [1]. This assumption is also supported by the increase in PUFA and by the thrombogenic (TI) and the health-promoting (HPI) indexes. The TI was significantly affected by the dietary *Schizochytrium* spp. inclusion, even for the low dietary level.

### 4.3. Oxidative Status

The antioxidant defense mechanisms are triggered and activated in conditions of oxidative stress, which is utterly attributed to the imbalance of free radical production. Moreover, since *Schizochytrium* spp. is rich in PUFA, this could suggest the increased susceptibility of the organism and consequently its products to oxidation. However, to the best of our knowledge, there are only a few studies dealing with the oxidative impact of dietary *Schizochytrium* spp.

Notwithstanding, the MDA is a major product of lipid peroxidation in cells [20], epitomizes a precise index for assessing PUFA peroxidation, and is one of the most commonly applied biomarkers for oxidative stress in a plethora of human health problems [49]. The rise of the MDA is in accordance with previous studies in *Schizochytrium* spp. fed goats [27] and in DHA-rich fed cows [50]. These results further support the assumption that the MDA rise could be since they are susceptible to peroxidation [17].

Correspondingly, the PC measurements are used as an accepted biomarker for protein oxidation. The increase in PC in blood plasma is widely linked to human health problems [51]. As far as the formation of PCs is concerned, it could be attained either from the activity of ROS on the protein side chain or indirectly by the secondary effects of lipid peroxidation [52], although the PCs created from lipid-derived aldehydes are more ubiquitous than those derived from the oxidation of the amino acid chain [53]. The increase in the PC content in dairy ewes is also reported as a result of the dietary inclusion of soya bean and fish oil [26]. The increase exemplified for both MDA and PC content might indicate the presence of oxidative stress. In view of the fact that PUFA may elude the rumen biohydrogenation process, they turn out to be superior targets for free radicals. For instance, lipid peroxidation occurred in the heart and liver of rats provided with 160 g fish oil blend/kg [22] while the counterpart arose with the inclusion of an 80 g fish oil blend/kg diet [21].

The ABTS assay performed in blood plasma for the evaluation of the antioxidant status of the animals strengthens our idea for the activation of the antioxidant mechanisms in order for the organism to deal with oxidative stress. For the highest level of microalgae incorporation (SC40), it has clearly been illustrated that a scavenging activity for ROS has been activated, as the results of the ABTS assay indicate not only for the dietary inclusion but also for the interaction of the factors.

The major ROS that can cause lipid peroxidation is that of OH*, which is created by the Fenton reactions. Catalase is an enzyme involved in degrading/detoxificating the peroxides [54]. A trend of higher CAT activity which was observed in the SC30 and SC40 ewes, confirms its protective role against lipid peroxidation as a cellular response to oxidative stress. Another important enzyme for the antioxidant system is GR which acts as a catalytic agent for the restoration of active reduced GSH, the main cellular antioxidant. Likewise, GST not only detoxifies electrophilic compounds but also performs the conjunction of them, and in this way decreasing their detrimental effects [55]. So, the enhancement of the activity for the higher dietary levels (SC30 and SC40) is related to the high concentration of MDA. This is also in consensus with the results from Mavrommatis et al. [23]. In contrast to our findings, an increase in SOD activity in the blood plasma of goats fed with 40 g or 60 g *Schizochytrium* spp./day was found which might indicate species differences [23].

The total antioxidant capacity and the enzymatic activities of milk were not substantially modified by the diets. However, only numerical enhancements on the activities of SOD and CAT were observed in the SC40 group where the MDA concentration depicted its significant peak. Although the inclusion of *Schizochytrium* spp. induced a low-grade oxidative stress in the milk of SC30 fed ewes, the MDA concentration was not affected while PCs were suppressed indicating that the organism’s native antioxidant defense together with the microalga antioxidant compounds were able to neutralize the oxidative burden. The same has been described in goats fed with whole sesame seeds, also rich in PUFA [56]; in the study by Mavrommatis et al. [23] the PCs in milk of goats were increased only when supplemented with 60 g/kg of *Schizochytrium* spp.

## 5. Conclusions

The incorporation of microalgae in ruminants’ diets aims to improve the fatty acids profile of milk and dairy products. In our study, the supplementation of *Schizochytrium* spp. in ewes’ diets clearly demonstrated these advantages in the view of the enhancement of the beneficial CLA, DPA and DHA. All three inclusion levels confirmed these beneficial features of *Schizochytrium* spp., although fat reduction occurred for the high inclusion levels. Moreover, the inclusion of 30 g and 40 g clearly affected the oxidative stability of the organism. Hence, it is more appropriate for the diets to be supplemented with 20g of *Schizochytrium* spp. as this is better for milk fat preservation, the oxidative status of the animals and the oxidative stability of the products. However, it could be prudent to use a combination of *Schizochytrium* spp. as a high PUFA feed supplement with natural antioxidant compounds for improving the oxidative stability of dairy products. This may be a target for future research to be done in order to produce PUFA-enriched dairy products with high oxidative stability.

## Figures and Tables

**Table 1:** Main characteristics of diets (CON, SC20, SC30, and SC40) consumed by ewes involved in the trial. *Concentrate composition (g/kg), ration components consumption (g/kg) and Ingredients of concentrate (g/Kg), ration components (Kg/ewe/day) and chemical composition (g/day) of the diets were administered to the four groups (CON, SC20, SC30, and SC40) of ewes involved in the trial*.

	**CON**		**SC20**		**SC30**			**SC40**	
**Concentrates Composition (g/kg)**									
Maize grain	472		477		467			462	
Sunflower meal	80		80		80			80	
Wheat middlings	70		70		70			70	
Barley grain	100		100		100			100	
Soyabean meal	185		185		185			185	
Vitamin and mineral premix	25		25		25			25	
Limestone	14		14		14			14	
Sodium bicarbonate	3		3		3			3	
Mycotoxin binder	1		1		1			1	
Soyabean oil	5		-		-			-	
Yeast product	25		25		25			25	
Palm oil	20		-		-			-	
*Schizochytrium* spp.	-		20		30			40	
**Consumption of Ration Components (kg/ewe/day)**									
Control concentrate mix	1.83		0.83		0.83			0.83	
Treated concentrate mix	-		1		1			1	
Alfalfa hay	1.83		1.83		1.83			1.83	
**Feeds Chemical Composition (g/kg)**									
	CON	SC20	SC30	SC40			Alfalfa hay		*Schizochytrium* spp.
Dry matter	931.3	929.5	918.6	934.5			925.5		980.0
Ash	62.6	67.9	69.4	67.1			72.0		88.0
Crude protein	151.7	152.2	155.0	156.4			216.3		167.0
Ether extract	32.5	32.0	32.6	36.3			13.0		520.0
Ash-free NDF treated with amylase	168.0	175.2	167.0	171.0			517.0		0
Acid Detergent Fiber	77.0	82.3	76.6	77.7			332.0		0
Non-Fibrous Carbohydrate	583.4	572.7	576.0	569.2			181.7		225.0
**Fatty Acids Profile (%)**									
C_14:0_	0.53	1.52	2.23	2.58			6.2		4.3
C_16:0_	35.74	16.36	16.49	16.49			36.77		11.2
C_18:0_	3.90	2.16	1.88	1.73			2.33		0.2
C_18:1 cis-9_	17.07	18.90	16.19	15.67			2.49		-
C_18:2n6c_	38.90	41.84	36.08	32.80			18.27		-
C_18:3n3_	2.80	2.65	2.42	2.18			30.68		0.1
C_20:5n3_	0.03	0.28	0.37	0.43			-		0.5
C_22:5n6_	0.02	4.25	6.46	7.50			-		7.9
C_22:6n3_	0.00	10.57	16.25	18.91			-		21.8
C_24:0_	0.18	0.24	0.23	0.22			-		0.1
**Daily Nutrients Intake (g/ewe)**									
	**CON**		**SC20**			**SC30**			**SC40**
Dry matter	3398		3396			3385			3401
Ash	246		252			253			251
Crude protein	677		675			678			680
Ether extract	83		83			83			87
Ash-free NDF treated with amylase	1254		1261			1253			1257
Acid Detergent Fiber	748		754			748			749
Non-Fibrous Carbohydrate	1400		1389			1393			1386
C_14:0_	1.8		2.1			2.3			2.6
C_16:0_	30.0		23.6			23.8			24.4
C_18:0_	2.9		2.3			2.2			2.2
C_18:1 cis-9_	10.7		11.2			10.5			10.9
C_18:2n6c_	27.5		28.2			26.6			26.7
C_18:3n3_	9.0		8.9			8.8			8.8
C_20:5n3_	0.0		0.1			0.1			0.2
C_22:5n6_	0.0		1.4			2.1			2.7
C_22:6n3_	0.0		3.4			5.3			6.9
C_24:0_	0.1		0.1			0.1			0.1

NDF = Neutral detergent fiber; CON = control concentrate without microalgae; SC20 = control concentrate with 20 g *Schizochytrium* spp./ewe/day; SC30 = control concentrate with 30 g *Schizochytrium* spp./ewe/day; SC40 = control concentrate with 40 g *Schizochytrium* spp./ewe/day.

**Table 2:** Milk yield and milk chemical composition of ewes fed diets (CON, SC20, SC30, SC40) with different levels of *Schizochytrium* spp. microalgae (20, 30 and 40 g) throughout the experimental period (10, 20, 30, 40, 50 experimental days).

	Diet (D)					Time (T)						Effect		
	CON	SC20	SC30	SC40	SEM	10	20	30	40	50	SEM	D	T	D×T
Milk yield (g)	1624	1497	1658	1614	142.66	1478 ^a^	1539 ^ab^	1693 ^c^	1720 ^bc^	1562 ^abc^	88.80	NS	NS	NS
FCM_6%_	1736	1523	1578	1368	168.23	1413 ^a^	1498 ^ab^	1613 ^bc^	1716 ^c^	1514 ^abc^	103.52	NS	NS	NS
ECM	1585	1410	1461	1312	148.27	1328 ^a^	1386 ^ab^	1511 ^bc^	1576 ^c^	1409 ^abc^	91.04	NS	NS	NS
Fat (%)	6.40 ^A^	5.89 ^AB^	5.53 ^BC^	5.05 ^C^	0.239	5.45 ^a^	5.75 ^abc^	5.88 ^bc^	5.89 ^b^	5.62 ^ac^	0.159	**	*	NS
Fat yield (g)	105.21	90.64	91.60	80.63	11.10	83.15 ^a^	88.43 ^ab^	97.98 ^b^	102.22 ^b^	88.31 ^ab^	6.70	NS	NS	NS
Protein (%)	6.70 ^A^	6.53 ^AB^	6.18 ^B^	6.31 ^AB^	0.183	6.52 ^ab^	6.33 ^c^	6.71 ^a^	6.24 ^c^	6.35 ^bc^	0.107	*	***	NS
Protein yield (g)	107.70	98.68	100.98	99.39	10.13	95.36 ^a^	96.22 ^ac^	111.08 ^b^	107.63 ^bc^	98.15 ^ac^	6.04	NS	*	NS
Lactose (%)	4.50	4.60	4.67	4.58	0.060	4.59 ^a^	4.55 ^a^	4.56 ^a^	4.56 ^a^	4.68 ^b^	0.039	NS	*	NS
TS (%)	18.21 ^A^	17.58 ^AB^	16.90 ^BC^	16.40 ^C^	0.329	17.09 ^a^	17.17 ^a^	17.67 ^b^	17.22 ^a^	17.20 ^a^	0.193	**	t	NS
SNF (%)	11.96	11.89	11.64	11.65	0.177	11.86 ^ab^	11.65 ^cd^	12.03 ^a^	11.57 ^d^	11.81 ^bc^	0.102	NS	***	NS

SEM: Standard error of means; Means with different superscripts in each row between the four diets (A, B, C, D) and between the five-sampling time (a, b, c, d), for each parameter differ significantly; t < 0.1 = trend, * *p* < 0.05, ** *p* < 0.01, *** *p* < 0.001; CON = control concentrate without microalgae; SC20 = control concentrate with 20 g *Schizochytrium* spp./ewe/day; SC30 = control concentrate with 30 g *Schizochytrium* spp./ewe/day; SC40 = control concentrate with 40 g *Schizochytrium* spp./ewe/day; FCM: Fat corrected milk in 6% according to the equation Y6% = (0.28 + 0.12F) M, where F = fat% and M = milk yield in g; ECM: Energy corrected milk = milk yield × (0.071 × fat (%) + 0.043 × protein (%) + 0.2224).

**Table 3:** The mean individual fatty acids (FA) (% of total FA), FA groups, and health indexes of milk of ewes fed diets (CON, SC20, SC30, SC40) with different levels of *Schizochytrium* spp. microalgae (20, 30 and 40 g) throughout the experimental period (10, 20, 30, 40, 50 experimental days).

	Diets (D)					Time (T)						Effect		
	CON	SC20	SC30	SC40	SEM	10	20	30	40	50	SEM	D	T	D×T
**C_4:0_**	3.55	3.82	3.98	3.78	0.242	3.25 ^a^	3.99 ^bc^	3.84 ^bc^	3.99 ^b^	3.83 ^c^	0.133	NS	***	***
**C_6:0_**	2.92	2.96	3.08	2.98	0.121	2.95 ^ac^	3.13 ^b^	3.00 ^abc^	3.01 ^c^	2.84 ^a^	0.077	NS	*	*
**C_8:0_**	2.60	2.76	2.91	2.71	0.165	2.97 ^a^	2.84 ^ab^	2.72 ^bcd^	2.68 ^c^	2.51 ^d^	0.098	NS	**	NS
**C_10:0_**	8.37	8.60	9.01	8.32	0.569	9.90 ^a^	8.77 ^b^	8.43 ^bcd^	8.17 ^c^	7.59 ^d^	0.321	NS	***	*
**C_11:0_**	0.00	0.02	0.02	0.01	0.008	0.08 ^a^	0.00 ^b^	0.00 ^b^	0.01 ^b^	0.00 ^b^	0.006	NS	**	NS
**C_12:0_**	5.05	5.44	5.40	5.16	0.253	6.32 ^a^	5.35 ^b^	5.15 ^bcd^	4.88 ^c^	4.61 ^d^	0.157	NS	***	NS
**C_13:0_**	0.01	0.02	0.01	0.01	0.007	0.06 ^a^	0.00 ^b^	0.00 ^b^	0.00 ^b^	0.00 ^b^	0.004	NS	**	NS
**C_14:0_**	13.06	13.86	14.38	14.40	0.421	14.90 ^a^	14.07 ^b^	13.78 ^bcd^	13.67 ^c^	13.20 ^d^	0.258	NS	***	NS
**C_14:1_**	0.44	0.41	0.43	0.43	0.039	0.40	0.47	0.43	0.41	0.41	0.026	NS	NS	NS
**C_15:0_**	1.02	1.03	0.96	0.95	0.059	1.08 ^a^	0.96 ^b^	0.97 ^b^	0.99 ^ab^	0.96^b^	0.035	NS	t	NS
**C_15:1_**	0.19	0.15	0.13	0.17	0.037	0.21 ^a^	0.19 ^ab^	0.11 ^c^	0.15 ^bc^	0.14 ^bc^	0.024	NS	*	NS
**C_16:0_**	28.74	28.13	30.36	30.90	0.800	30.16 ^a^	29.46 ^ab^	28.71 ^b^	29.62 ^ab^	29.71 ^a^	0.460	NS	t	*
**C_16:1_**	1.10	1.15	1.14	1.24	0.071	1.12 ^ab^	1.23 ^c^	1.20 ^ac^	1.12 ^b^	1.11 ^b^	0.041	NS	t	NS
**C_17:1_**	0.14 ^A^	0.12 ^AB^	0.04 ^B^	0.16 ^A^	0.033	0.16 ^a^	0.08 ^b^	0.20 ^ab^	0.06 ^b^	0.09 ^b^	0.028	t	**	NS
**C_18:0_**	7.47 ^A^	4.28 ^B^	2.46 ^C^	1.72 ^C^	0.600	3.13 ^a^	3.28 ^a^	4.70 ^bc^	4.07 ^b^	4.74 ^c^	0.383	***	***	NS
**C_18:1 trans_**	0.61 ^A^	2.88 ^B^	3.49 ^B^	4.02 ^B^	0.600	1.53 ^a^	3.84 ^bc^	2.19 ^ac^	3.08 ^b^	3.12 ^bc^	0.445	**	*	NS
**C_18:1 t11_**	0.82 ^A^	3.28 ^B^	4.59 ^BC^	5.19 ^C^	0.558	4.13	3.13	3.33	3.36	3.42	0.415	***	NS	*
**C_18:1 c9_**	18.51 ^A^	13.19 ^B^	9.12 ^C^	8.24 ^C^	0.838	10.31 ^a^	11.16 ^b^	13.46 ^cd^	12.57 ^c^	13.81 ^d^	0.558	***	***	*
**C_18:1 c11_**	0.60 ^A^	0.76 ^B^	0.70 ^AB^	0.76 ^B^	0.043	0.67	0.74	0.70	0.70	0.71	0.027	t	NS	*
**C_18:2n6t_**	0.46 ^A^	0.73 ^B^	0.67 ^B^	0.66 ^B^	0.038	0.46 ^a^	0.60 ^b^	0.69 ^c^	0.76 ^d^	0.65 ^bc^	0.026	***	***	***
**C_18:2n6c_**	2.65 ^A^	2.34 ^B^	2.06 ^C^	2.00 ^C^	0.086	2.10 ^a^	2.18 ^a^	2.34 ^b^	2.27 ^ab^	2.43 ^b^	0.065	***	*	NS
**C_20:0_**	0.16 ^A^	0.09 ^B^	0.06 ^B^	0.05 ^B^	0.019	0.07 ^a^	0.12 ^b^	0.07 ^a^	0.14 ^c^	0.04 ^a^	0.013	**	***	NS
**C_18:3n3_**	0.46	0.45	0.43	0.35	0.039	0.42 ^a^	0.34 ^b^	0.43 ^a^	0.40 ^a^	0.54 ^c^	0.024	NS	***	***
**C_18:2 c9, t11_**	0.53 ^A^	2.19 ^B^	2.36 ^B^	3.13 ^B^	0.325	2.02 ^ab^	2.25 ^a^	1.89 ^b^	2.16 ^ab^	1.93 ^b^	0.193	***	*	NS
**C_18:2 t10, c12_**	0.02 ^A^	0.24 ^B^	0.38 ^C^	0.53 ^D^	0.046	0.22^a^	0.43 ^b^	0.31 ^c^	0.34 ^c^	0.17 ^a^	0.030	***	***	***
**C_20:3n3_**	0.24 ^A^	0.33 ^B^	0.39 ^C^	0.42 ^C^	0.016	0.30 ^a^	0.34 ^b^	0.37 ^b^	0.37 ^b^	0.36 ^b^	0.011	***	**	***
**C_20:5_**	0.01 ^A^	0.01 ^A^	0.04 ^AB^	0.08 ^B^	0.014	0.03	0.02	0.01	0.05	0.04	0.012	*	NS	***
**C_24:1_**	0.00 ^A^	0.08 ^A^	0.34 ^B^	0.47 ^C^	0.038	0.26 ^a^	0.24 ^a^	0.20 ^b^	0.21 ^b^	0.20 ^b^	0.021	***	**	***
**C_22:5n6_**	0.01 ^A^	0.03 ^AB^	0.08 ^B^	0.17 ^C^	0.020	0.11 ^a^	0.07 ^bc^	0.05 ^b^	0.04 ^b^	0.11 ^ac^	0.015	***	**	**
**C_22:6n3_**	0.00 ^A^	0.50 ^B^	0.87 ^C^	1.06 ^D^	0.065	0.55 ^a^	0.60 ^b^	0.62 ^b^	0.61 ^b^	0.62 ^b^	0.035	***	t	***
**SCFA**	17.29	18.19	19.07	17.94	0.712	19.18 ^a^	18.76 ^b^	18.02 ^bcd^	17.88 ^c^	16.78 ^d^	0.461	NS	***	NS
**MCFA**	47.89 ^A^	48.55 ^AB^	51.16 ^B^	51.34 ^B^	0.920	52.56 ^a^	49.84 ^b^	48.63 ^bc^	49.17 ^bc^	48.48 ^c^	0.601	*	***	*
**LCFA**	7.63 ^A^	4.37 ^B^	2.53 ^C^	1.77 ^C^	0.606	3.20 ^a^	3.40^a^	4.78 ^bc^	4.22 ^b^	4.78 ^c^	0.385	***	***	NS
**MUFA**	22.40	22.06	20.09	20.66	0.745	18.82 ^a^	21.13 ^b^	21.85 ^bc^	21.68 ^b^	23.05 ^c^	0.535	NS	***	**
**PUFA**	4.35 ^A^	6.87 ^B^	7.32 ^B^	8.45 ^C^	0.364	6.25 ^a^	6.86 ^b^	6.72 ^ab^	7.03 ^b^	6.88 ^b^	0.222	***	t	NS
**SFA**	73.11	71.08	72.61	70.94	0.914	74.92 ^a^	72.00 ^b^	71.43 ^bc^	71.28 ^b^	70.05 ^c^	0.637	NS	***	**
**UFA**	26.88	28.92	27.38	29.05	0.914	25.07 ^a^	27.99 ^b^	28.56 ^bc^	28.71 ^b^	29.94 ^c^	0.637	NS	***	**
**SFA/UFA**	2.74	2.55	2.72	2.51	0.109	3.04 ^a^	2.61 ^b^	2.59 ^bc^	2.52 ^b^	2.39 ^c^	0.076	NS	***	**
**AI**	3.23	3.20	3.50	3.31	0.150	3.91 ^a^	3.30 ^b^	3.24 ^bc^	3.14 ^b^	2.96 ^c^	0.103	NS	***	**
**TI**	3.26 ^A^	2.80 ^B^	2.74 ^B^	2.63 ^B^	0.105	3.22 ^a^	2.89 ^b^	2.78 ^bc^	2.76 ^bc^	2.64 ^c^	0.072	**	***	***
**HPI**	0.31	0.32	0.26	0.28	0.018	0.24 ^a^	0.28 ^b^	0.32 ^bc^	0.30 ^b^	0.32 ^c^	0.013	NS	***	NS

Means with different superscripts in each row between the four diets (A, B, C, D) and between the five-sampling time (a, b, c, d), for each parameter differ significantly (*p* ≤ 0.05); t < 0.1 = trend, * *p* < 0.05, ** *p* < 0.01, *** *p* < 0.001; SEM: Standard error of means; CON = control concentrate without microalgae; SC20 = control concentrate with 20 g *Schizochytrium* spp./ewe/day; SC30 = control concentrate with 30 g *Schizochytrium* spp./ewe/day; SC40 = control concentrate with 40 g *Schizochytrium* spp./ewe/day.

**Table 4:** Enzyme activities (Units/mL), total antioxidant capacity and oxidative status biomarkers in blood plasma and milk of ewes fed diets (CON, SC20, SC30, SC40) with different levels of *Schizochytrium* spp. microalgae (20, 30 and 40 g) throughout the experimental period (10, 20, 30, 40, 50 experimental days).

	**Diet (D)**					**Time (T)**						**Effect**		
	CON	SC20	SC30	SC40	SEM	10	20	30	40	50	SEM	D	T	D×T
**Blood plasma**														
**MDA**	0.71 ^A^	0.85 ^B^	0.86 ^B^	0.84 ^B^	0.037	0.83	0.78	0.83	0.82	0.81	0.031	*	NS	NS
**PC**	3.09 ^A^	3.45 ^B^	3.62 ^BC^	3.84 ^C^	0.123	3.62 ^a^	3.36 ^b^	3.37 ^ab^	3.28 ^b^	3.87 ^c^	0.090	**	**	NS
**FRAP**	0.72	0.71	0.68	0.71	0.040	0.74 ^ab^	0.71 ^ab^	0.67 ^ab^	0.66 ^a^	0.74 ^b^	0.033	NS	t	NS
**ABTS**	48.94 ^A^	49.11 ^A^	49.28 ^A^	51.38 ^B^	0.428	46.96 ^a^	49.04 ^b^	50.62 ^c^	51.20 ^c^	50.56 ^c^	0.353	**	***	***
**CAT**	42.92 ^A^	38.26 ^A^	66.88 ^B^	50.34 ^AB^	7.602	38.68 ^a^	39.18 ^a^	52.17 ^b^	60.29 ^b^	57.69 ^b^	4.775	t	**	NS
**SOD**	13.48	13.74	12.49	12.05	0.579	14.08 ^a^	13.12 ^a^	11.53 ^b^	12.88 ^a^	13.10 ^a^	0.488	NS	*	**
**GSH-Px**	0.18	0.19	0.16	0.19	0.009	0.19 ^a^	0.17 ^ab^	0.16 ^b^	0.18 ^a^	0.19 ^a^	0.007	NS	***	***
**GST**	0.06 ^A^	0.06 ^AC^	0.09 ^B^	0.08 ^BC^	0.007	0.06 ^a^	0.06 ^a^	0.08 ^b^	0.08 ^b^	0.08 ^b^	0.005	*	**	NS
**GR**	0.03 ^A^	0.04 ^B^	0.03 ^AB^	0.03 ^AB^	0.002	0.03 ^ab^	0.04 ^c^	0.04 ^c^	0.03 ^a^	0.03 ^b^	0.001	t	***	NS
**Milk**														
**MDA**	0.41 ^A^	0.51 ^BC^	0.48 ^AB^	0.58 ^C^	0.027	0.48 ^ab^	0.51 ^a^	0.49 ^ab^	0.53 ^a^	0.45 ^b^	0.021	**	*	NS
**PC**	3.65 ^A^	3.24 ^AB^	2.99 ^B^	3.39 ^AB^	0.139	3.07 ^ac^	4.07 ^b^	3.33 ^a^	3.28 ^ac^	2.84 ^c^	0.152	*	**	NS
**FRAP**	3.71	3.34	2.97	3.10	0.236	4.71 ^a^	3.84 ^b^	3.34 ^b^	2.23 ^c^	2.27 ^c^	0.192	NS	***	NS
**ABTS**	42.61	44.58	40.86	44.15	1.933	53.96 ^a^	47.58 ^b^	43.16 ^b^	32.17 ^c^	38.36 ^d^	1.844	NS	***	NS
**CAT**	3.83	3.88	4.13	4.38	0.415	4.02	3.89	3.84	4.15	4.36	0.274	NS	NS	*
**SOD**	20.25	20.53	20.54	22.04	1.835	18.27 ^a^	16.86 ^a^	20.15 ^a^	24.54 ^b^	24.37 ^b^	1.367	NS	**	*
**GSH-Px**	0.57	0.44	0.48	0.47	0.068	0.34 ^a^	0.42 ^b^	0.60 ^c^	0.72^c^	0.39 ^ab^	0.041	NS	***	NS

Means with different superscripts in each row between the four diets (A, B, C, D) and between the five-sampling time (a, b, c, d), for each parameter differ significantly (*p* ≤ 0.05) t < 0.1 = trend, * *p* < 0.05, ** *p* < 0.01, *** *p* < 0.001; SEM: Standard error of means; CON = control concentrate without microalgae; SC20 = control concentrate with 20 g *Schizochytrium* spp./ewe/day; SC30 = control concentrate with 30 g *Schizochytrium* spp./ewe/day; SC40 = control concentrate with 40 g *Schizochytrium* spp./ewe/day; MDA: Malondialdehyde (μΜ MDA); PC: Protein carbonyls (nmol/mL); CAT: Catalase (Units/mL); FRAP: Ferric Reducing Ability of Plasma (μΜ Ascorbic acid); ABTS: 2,2′-azino-di (3-ethylbenzthiazoline-6-sulfonic acid (% inhibition); SOD: Superoxide dismutase (Units/mL); GSH-Px: Glutathione peroxidase (Units/mL); GST: Glutathione transferase (Units/mL); GR: Glutathione reductase (Units/mL).

## Data Availability

Data are contained within the article.
